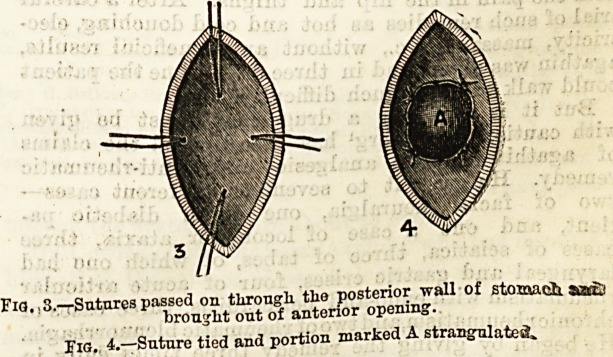# Surgery of the Stomach

**Published:** 1894-01-13

**Authors:** 


### SURGERY OF THE STOMACH.

The fearful mortality (probably 95 per cent. at least) attending a perforating gastric ulcer has brought this malady into the domain of surgery, in the hope that early operative interference may save the patient from this most fatal condition. Mr. Gilbert Barling records[1] three cases, one of which was successful; and Mr. Haslam has also reported[2] a successful case. Haslam thinks the best position for the abdominal incision is above the umbilicus, and to the left of the mid line, so as to miss the falciform ligament. He draws attention to the fact that the anatomically anterior surface of the stomach may vary in position according to the state of distension of that organ, and that, therefore, the site of the ulceration may be farther removed from the abdominal incision than operators might expect. The abdomen being opened positive evidence may be at once found in the presence of free gas or food. Mr. Barling does not think it well to dogmatise on the method of procedure, but gives the following as the main outlines: The area between the stomach and liver should be examined with care, so that any localised collection here should, if possible, be prevented from soiling the remainder of the cavity.

The perforation should be sought for on the anterior wall, especially towards the lesser curvature, where it is most commonly situated. If found, the perforation should be sutured by the continuous Lembert suture, the stomach being brought out of the wound, if this is possible. Paring the edges of the ulcer is generally condemned as taking up too much time. If the ulcer cannot be sutured, it may possibly be stitched to the lower angle of the incision, but failing this the only resource is drainage, with a tube leading directly to the point of leakage.

Whether suturing be possible or not, it is of the greatest importance to thoroughly flush the abdominal cavity with hot water. When this has been done, the drain should be placed *in situ* close to the point of perforation, whether it has been sutured or not, and it is desirable to introduce another tube through an incision in the lower part of the abdominal wall, so as to drain the pelvic cavity.

The operation of pyloroplasty for non-malignant stenosis of the pylorus has been very successful, and appears to be an important advance upon Loreta's method of divulsion of the pylorus. Mr. Gould refers[3] to twenty-three cases, and of these sixteen recovered from the operation, five died from it, and in two the result is not stated. This gives a mortality of just under 25 per cent. But the operation is even more favourable than this, for Mr. Gould omitted the case of Drs. Limont and Page[4], which was the first operation of this kind performed in England, and since then Dr. Lange has published[5] his third case, and all of these were successful. The mortality of Loreta's operation is about 40 per cent. Of the sixteen cases of pyloroplasty reported as recovered two died shortly afterwards (two months and five months) from tubercular disease, but Heineke's first patient was known to be well four years after operation. The statistics of Loreta's operation show cases of death from complete rupture of the pylorus on its posterior aspect, and also from hæmorrhage; the "plastic" operation is entirely free from these dangers. Divulsion has been followed by recurrence of the stricture, and in many cases the operation has been repeated; and looking at analogous cases (such as dilatation of the sphincter ani, or sudden dilatation of a strictured urethra or rectum) is this what one might expect. Pyloroplasty, on the other hand, introduces new and presumably healthy tissue into the pyloric ring—tissue with no tendency to contract.

It is well known that adhesions in the abdominal cavity may give rise to serious symptoms, but it is not generally recognised that dilatation of the stomach may be cured by their separation—an operation obviously less risky than that of pyloroplasty. Mr. Mayo Robson has reported[6] two such cases, both of which were successful. In one case the original cause of the adhesions was gall-stones, and in the other ulceration of the stomach. In both cases medical treatment had failed, and Mr. Robson stated that he thought it would be desirable in all cases of obscure abdominal pain, after medical treatment had been fully tried and failed, to open the abdomen in order to clear up the diagnosis, and then to adopt that line of treatment which seemed to be indicated, and it was his impression in gall-stone cases that more benefit accrued from separation of adhesions than from removal of stones. The obvious disadvantage of this method of treatment is that the adhesions are apt to re-form, although this is probably prevented by the return of the viscera to their normal positions after the adhesions have been separated.

Pylorectomy for carcinoma of the pylorus, where the growth is not fixed and can be fairly easily removed, seems to give a longer term of life than gastro-enterostomy, although, of course, the final outlook is not very hopeful, as the disease nearly always recurs. Successful cases have been reported by Dr. Lange[7] and M. Polosson[8].

In the performance of gastrostomy for malignant disease of the œsophagus we must select that operation which seems best suited to the case. The great trouble the surgeon has to contend with is leakage through the incision in the stomach. It appears that Witzel's operation seems least likely to be followed by leakage, and has therefore been advocated[9] by Dr. Meyer. The essential steps of the operation are as follows: An incision is made parallel to the left border of the ribs with blunt division of that portion of the rectus muscle which is in the line of the incision. Division of the peritonæum. Primary incision of the stomach by a very small hole. Into this opening a snugly-fitting rubber tube is at once introduced, and then buried in the wall of the stomach to the extent of from one to one and a-half inches, by stitching over it two folds of the stomach wall. These folds run from the left down to the right end upward. The entire area is then stitched to the peritoneal wound by interrupted sutures, thus rendering the operative field extra-peritoneal, and the abdominal wound is closed. Witzel had thus operated on two patients for stricture of the œsophagus, and in neither case was there any leakage, although the patients were fed through the tube immediately after the operation. Witzel explains this fact by assuming that a valve-like occlusion was most probably formed at the inner opening of the fistula by the mode of burying the tube. The operation takes some time in its performance, and can therefore only be done in selected cases. Frank[10] has also devised a method of gastrostomy, for which he claims an efficient closure of the fistulous opening. The operation consists in making an incision parallel to the costal arch, drawing forward a 4 cm. flap of the anterior wall of the stomach, and uniting the base of this to the serous surface. Then a 2 cm. incision is made through the skin at the upper part of the costal arch. Between this and the first incision is a bridge of skin, under which a suture is passed, and the stomach flap is drawn under this flap of skin and fixed to the opening at the upper part of the costal arch. The wound at the lower part of the costal arch is then closed.

Cases of gastro-enterostomy by means of Senn's decalcified bone-plates seem to show that the inosculation is at first quite satisfactory, that for some six or eight weeks it remains fairly free, and that then it slowly contracts with the recurrence of symptoms of obstruction. This is probably due to the fact that a clean incision in the stomach, involving no loss of tissue, tends to heal remarkably well. To obviate this tendency to contraction, Mr. Paul[11] has devised a modification of Senn's method, which strangulates the connected surfaces of the stomach and intestines, producing by sloughing a clean circular opening between the bowel and the back of the stomach, which in experiments on dogs has shown no tendency to diminish up to a period of 107 days, the longest experiment made. The special apparatus required involves nothing more than a hard ring, preferably of bone, about three-quarters of an inch in diameter for human intestine, and perforated with four small equidistant holes. The ring may be rounded on all sides or only on one, as in the diagram (Fig. 1). This surface should always be round in order that it may not cut the piece out too sharply and lessen the breadth of surrounding adhesions. The four holes are charged each with a needle carrying a strong double silk thread, very securely double-knotted on the under side. The operation is performed as follows: The abdomen is opened, and the first part of the jejunum is found and brought out of the wound in the usual way. A small incision is made into the bowel, where it can be applied to the lower and back part of the stomach without the least tension. Through this small wound the bone ring is slipped into the bowel, the needles are passed as in Fig. 2, and the opening is temporarily closed.

FIG. 1.—The bone ring charged with silk sutures and needles.

FIG. 2.—Bowel with ring and sutures in situ, and opening temporarily closed.

Next a cut of about an inch and a quarter in length is made in the front wall of the stomach opposite to the spot where the inosculation is desired, and the four needles are passed in regular order through the transverse meso-colon and posterior wall of the stomach, and are brought out of the front opening (Fig. 3). When they are drawn tight of course the intestine is

FIG. 3.—Sutures passed on through the posterior wall of stomach and brought out of anterior opening.

FIG. 4.—Suture tied and portion marked A strangulated.

firmly applied to the back of the stomach, and by cutting off the needles and tying the ligatures tightly, as in Fig. 4, the included discs of bowel and stomach are strangulated between the ring in the former and the ligatures in the latter. Whilst the parts are still held forwards by the ligatures the centre of this area may be cut out with a tenotomy knife, thus at once effecting a communication between the two viscera. Then the ligatures are cut short, the parts are allowed to drop back into position, and the opening in the front wall of the stomach is closed by a double row of fine green catgut sutures, by a continuous row in the mucous membrane, and by Lembert sutures in the outer coats. Finally the stomach is turned up, if possible, and a few Lembert sutures are applied on the outskirts of the inosculation to retain the parts in position when they lose the support of the ligatures by sloughing on the second day. These sutures are much more important in this than in Senn's operation; but if they cannot be used owing to fixation of the organ by cancerous infiltration—and it must be a very bad case in which none can be passed—the patient must be kept very still for at least a week. Such additional support is less urgently needed when the intestine is applied to the back than when it is applied to the front of the stomach, as the tension in the former case is much less, but it should never be neglected. The abdominal wound is always closed with deep sutures of fishing gut, including all the tissues from the peritoneum to the skin. Mr. Paul thinks the following conclusions are justified: (1) That the operation of gastro-enterostomy, when performed in this way with due precautions, is not more dangerous to life than with the decalcified bone plates; (2) that the new opening is in a better position; (3) that it will not spontaneously close; and (4) that it involves no unnatural displacement of the bowel in bringing it into contact with the stomach. Braun points out[12] that, after gastro-enterostomy, the contents of the stomach often pass into the proximal or pyloric limb of the attached loop of intestine, and as, in most instances, they cannot traverse the pylorus, they collect in the intervening portion of duodenum, distend it, and after a time regurgitate into the stomach through the fistula, causing much disturbance of digestion, vomiting, and exhaustion. This mishap, it is asserted, is a frequent cause of death after gastro-enterostomy. With the view of preventing this flow of the gastric contents into the proximal rather than into the distal portion of the intestinal canal, the author advocates the plan of performing, in the course of one operation, both gastro-enterostomy and intestinal anastomosis. In the latter stage a fistula is established between the two limbs of the loop of intestine that has been brought into communication with the stomach. If, after the operation, the food should tend to pass into the proximal portion of the loop, it will be forced by the peristaltic action of the stomach through the intestinal fistula, and reach the distal limb. The double operation is performed by the author in from one hour and a quarter to one and a half. He has had experience of this method in six cases, in three of these with success, in the remaining three with fatal results—death being due to exhaustion, and not to any gangrene or perforation at the seat of anastomosis, and occurring in the three cases on the second, fifteenth, and sixteenth days respectively after the date of operation.

[1] Brit. Med. Journ., June 17th, 1893. [2] Therapeutic Gazette, September, 1893. [3] Lancet, May 20th, 1893, p. 1183. [4] Lancet, July, 1892, and May 27th, 1893. [5] Annals of Surgery, August, 1893. [6] Lancet, October 21st, 1893. [7] Annals of Surgery, August, 1863. [8] Lyon Méd. September 24th, 1893. and Brit. Med. Journ., November 11th, 1893. [9] Annals of Surgery, May, 1893. [10] Wein. Klin. Woch, December 15th, 1892, and Brit. Med. Journ., January 14th, 1893. [11] Lancet, July 15th, 1893. [12] Arch. f. Klin. Chir, Bd. 45, and Brit. Med. Journ. April 1st, 1893.

## Figures and Tables

**Fig. 1. f1:**
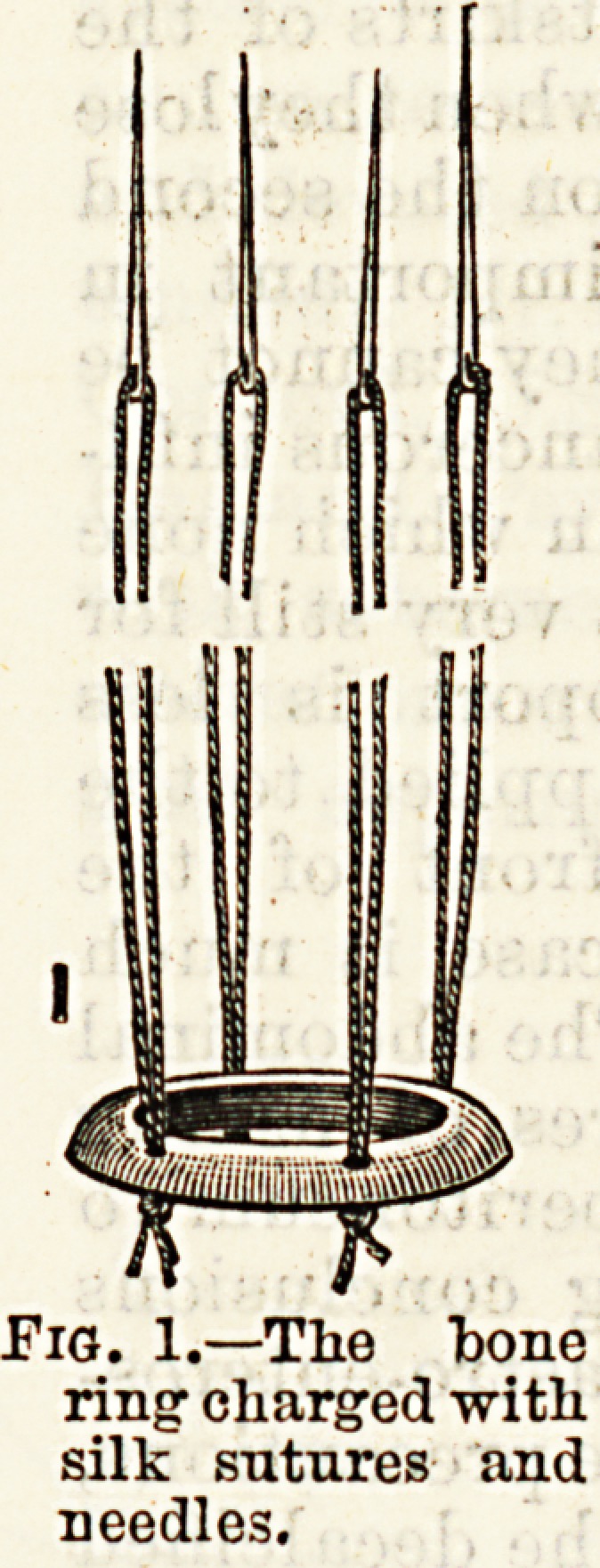


**Fig. 2. f2:**
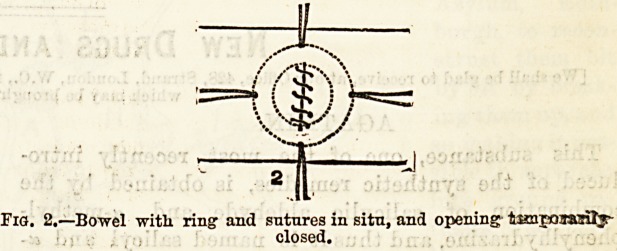


**Fig. 3. Fig. 4. f3:**